# Synthesis of MPB@ZnPc Nanomaterials and Their Application in the Treatment of Periodontitis

**DOI:** 10.3390/ijms27125161

**Published:** 2026-06-06

**Authors:** Qingyue Tan, Xuan Zhang, Yujuan Tian, Rui Li

**Affiliations:** School of Stomatology, Tianjin Medical University, Tianjin 300041, China; tqy18@tmu.edu.cn (Q.T.); zhangxuanzx@tmu.edu.cn (X.Z.)

**Keywords:** periodontitis, mesoporous Prussian blue, zinc phthalocyanine, photodynamic therapy, photothermal therapy, NF-κB signaling pathway

## Abstract

Periodontitis treatment remains challenging due to incomplete removal of plaque biofilms, increasing antibiotic resistance, and dysregulated host inflammatory responses. In this study, an MPB@ZnPc nanomaterial was constructed to achieve efficient antibacterial activity through the synergistic effects of photothermal therapy (PTT) and photodynamic therapy (PDT), while also exerting immunomodulatory functions under dark conditions. MPB@ZnPc (mesoporous Prussian blue @ zinc phthalocyanine) was synthesized using a polymer-templating method and systematically characterized. The results demonstrated that the nanomaterial exhibited excellent photothermal conversion efficiency and stability under near-infrared (NIR) irradiation. It also showed strong photocatalytic degradation performance toward methylene blue and rhodamine B, accompanied by substantial reactive oxygen species (ROS) generation. In vitro antibacterial assays revealed that MPB@ZnPc achieved significantly enhanced antibacterial efficacy compared with individual components, with bactericidal rates of 99.61 ± 0.52% against *Porphyromonas gingivalis* and 99.77 ± 0.32% against *Fusobacterium nucleatum*. The corresponding biofilm removal rates reached 93.60 ± 3.30% and 93.25 ± 3.30%, respectively. Under dark conditions, the nanomaterial exhibited good biocompatibility toward L929 cells and effectively inhibited lipopolysaccharide (LPS)-induced M1 polarization of macrophages, leading to reduced expression of pro-inflammatory cytokines, including *IL-1β*, *IL-6*, and *TNF-α*. Mechanistically, MPB@ZnPc suppressed the activation of the NF-κB signaling pathway. Overall, MPB@ZnPc provides a promising strategy for precise periodontitis treatment by integrating synergistic antibacterial activity with immunomodulatory effects.

## 1. Introduction

Periodontitis is a chronic inflammatory disease that, if left untreated, can cause irreversible damage to periodontal supporting tissues, including the gingiva, periodontal ligament, alveolar bone, and cementum, ultimately leading to tooth loss in severe cases [1,2]. *Porphyromonas gingivalis* (*P. gingivalis*) and *Fusobacterium nucleatum* (*F. nucleatum*) are recognized as key periodontal pathogens responsible for the initiation and progression of periodontitis [3]. Moreover, the extracellular polymeric substances (EPSs) secreted by these bacteria form a natural protective barrier, which hinders the penetration and efficacy of antimicrobial agents [4].

Conventional treatment strategies, primarily based on mechanical debridement combined with antibiotics, suffer from two major limitations. On the one hand, mechanical instruments are often unable to access deep periodontal pockets or complex anatomical structures, resulting in incomplete biofilm removal [5,6]. On the other hand, repeated antibiotic use can induce bacterial resistance, significantly reducing therapeutic efficacy [7]. Therefore, developing novel antibacterial strategies capable of efficiently eliminating biofilms while minimizing the risk of resistance has become a critical challenge in periodontitis treatment.

Nanomaterial-based therapies mediated by near-infrared (NIR) light have emerged as promising alternatives for antibacterial applications. In particular, the combination of antibacterial photodynamic therapy (aPDT) and photothermal therapy (PTT) has attracted increasing attention due to its precise targeting, minimal invasiveness, and low propensity for inducing resistance [8,9]. Owing to its deep tissue penetration and spatiotemporal controllability, NIR light is considered an ideal excitation source for periodontal therapy [10]. aPDT relies on photosensitizers (PSs) to generate reactive oxygen species (ROS) under light irradiation, enabling efficient bacterial killing with low resistance potential [11]. However, the effectiveness of aPDT is highly dependent on oxygen availability, which limits its performance in hypoxic environments such as deep periodontal pockets [12,13]. In contrast, PTT converts absorbed light energy into localized heat through photothermal agents (PTAs), leading to bacterial ablation via thermal effects [14]. As a physical antibacterial approach, PTT is less likely to induce resistance and remains effective under anaerobic conditions [15]. Nevertheless, the high temperatures required for effective PTT may damage surrounding healthy tissues and potentially aggravate periodontal inflammation [16,17].

Given the complementary mechanisms of aPDT and PTT, integrating these two modalities into a single nanoplatform has been widely considered an effective strategy to overcome their individual limitations and enhance overall therapeutic efficacy. For instance, Yu et al. [18] developed an indocyanine green (ICG)-loaded MXene-based nanoplatform that combines the photothermal effect of MXene with the photodynamic effect of ICG, achieving efficient eradication of drug-resistant bacteria. However, most existing studies primarily focus on enhancing antibacterial performance through phototherapy, while relatively limited attention has been paid to the excessive host immune response and its regulation during periodontitis progression.

Importantly, the pathogenesis of periodontitis involves not only direct bacterial damage but also dysregulated host immune responses, which play a crucial role in periodontal tissue destruction [19,20]. In particular, the polarization of macrophages toward the pro-inflammatory M1 phenotype and the excessive release of inflammatory cytokines, such as *IL-1β*, *IL-6*, and *TNF-α*, are key contributors to disease progression [21,22]. Therefore, an ideal therapeutic strategy for periodontitis should simultaneously achieve effective antibacterial activity and immune regulation.

Prussian blue (PB) is known for its excellent photothermal conversion efficiency under NIR irradiation [23]. Notably, PB has been approved by the U.S. Food and Drug Administration (FDA) for the treatment of thallium and radioactive cesium poisoning, indicating its favorable biocompatibility [24,25]. Mesoporous Prussian blue (MPB), derived from PB with an ordered mesoporous structure, exhibits a larger surface area and enhanced loading capacity. Previous studies have demonstrated that MPB can inhibit M1 macrophage polarization, reduce inflammatory cytokine secretion, and suppress activation of the NF-κB signaling pathway [26]. NF-κB is a classical inflammation-related pathway closely associated with the onset and progression of periodontitis [27]. Under physiological conditions, NF-κB forms an inactive complex with its inhibitor IκB in the cytoplasm [28], whereas periodontal pathogens can activate this pathway, thereby exacerbating inflammatory responses [29].

Zinc phthalocyanine (ZnPc), a second-generation photosensitizer, exhibits strong absorption in the red-light region and a high singlet oxygen quantum yield, making it highly effective for aPDT applications [30]. In addition, previous studies have reported that ZnPc can reduce the secretion of pro-inflammatory cytokines, including *IL-1β*, *IL-6*, and *TNF-α*, by macrophages even under dark conditions [31].

In this study, we constructed a multifunctional nanoplatform (MPB@ZnPc) with both light-responsive antibacterial activity and immunomodulatory capability. Its photothermal and photodynamic properties under NIR irradiation, as well as its antibacterial efficacy against periodontal pathogens and biofilm removal capacity, were systematically evaluated. Furthermore, its anti-inflammatory activity under dark conditions and the underlying signaling pathways were preliminarily investigated. The results demonstrate that MPB@ZnPc achieves efficient bacterial eradication and biofilm disruption through the synergistic effects of PTT and aPDT under NIR irradiation, while suppressing excessive host inflammatory responses via immunomodulation in the absence of light. Collectively, this dual-functional nanoplatform offers a promising strategy for precise periodontitis therapy by integrating antibacterial and immunoregulatory effects.

## 2. Results

### 2.1. Material Characterization

**Figure 1:**
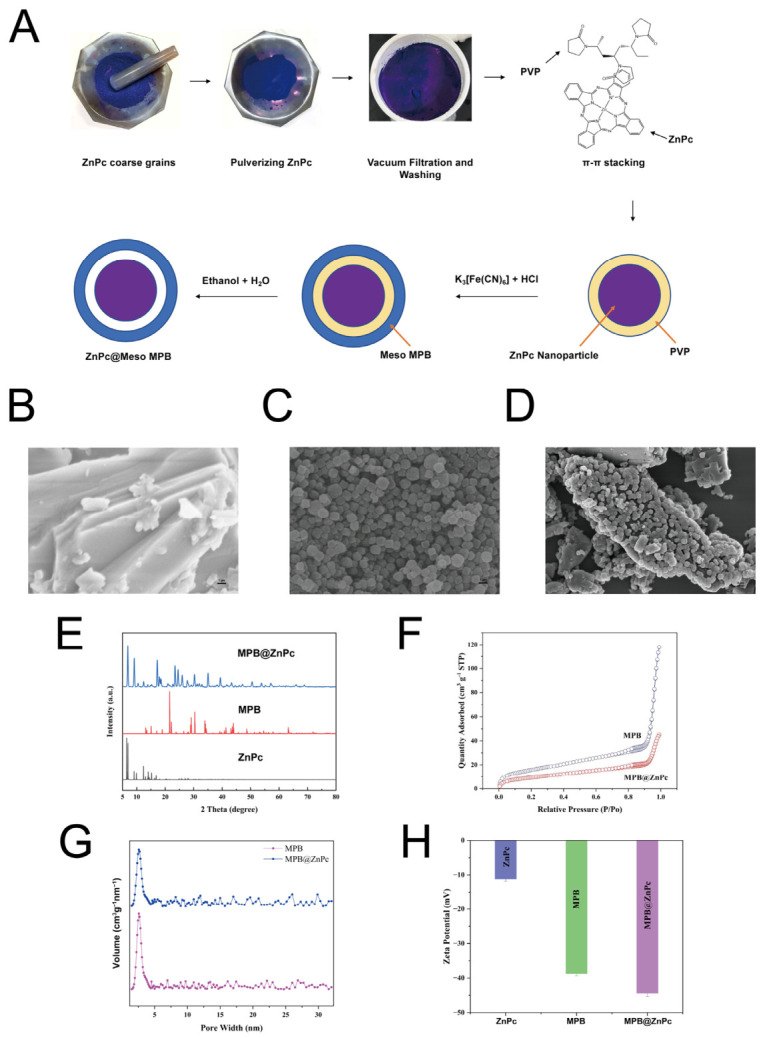
Material characterization. (**A**) Schematic illustration of the synthesis process of MPB@ZnPc. (**B**) SEM image of ZnPc (scale bar: 1 μm). (**C**) SEM image of MPB (scale bar: 1 μm). (**D**) SEM image of MPB@ZnPc (scale bar: 2 μm). (**E**) XRD patterns of ZnPc, MPB, and MPB@ZnPc. The blue, red, and gray curves represent MPB@ZnPc, MPB, and ZnPc, respectively. (**F**) N_2_ adsorption–desorption isotherms of MPB and MPB@ZnPc (blue line: MPB; red line: MPB@ZnPc). (**G**) Pore size distribution curves of MPB and MPB@ZnPc calculated by the BJH method. (**H**) Comparison of zeta potentials of MPB, ZnPc, and MPB@ZnPc (n = 3).

Figure 1B shows the SEM image of ZnPc. After co-grinding with NaCl followed by purification, ZnPc exhibited irregular flake-like and aggregated particulate structures with a broad size distribution, typically in the range of several hundred nanometers. This morphology is characteristic of organic small-molecule materials without specific dispersion treatment.

As shown in Figure 1C, MPB displayed uniform cubic nanoparticles with well-defined edges and a relatively narrow size distribution of approximately 100–150 nm. In contrast, the SEM image of MPB@ZnPc (Figure 1D) revealed that smaller cubic MPB nanoparticles were uniformly anchored onto the surface of larger flake-like ZnPc structures.

These morphological changes indicate the successful integration of ZnPc and MPB via the polymer-templating method, with MPB predominantly distributed on the surface of ZnPc.

The XRD patterns (Figure 1E) further confirmed the composite structure. Pure MPB exhibited characteristic diffraction peaks at 2θ = 21.3°, 30.7°, 34.8°, and 52.0°, while pure ZnPc showed additional peaks at 2θ = 6.9°, 9.1°, 12.5°, and 16.8°. Notably, MPB@ZnPc retained the characteristic peaks of both ZnPc (6.9°, 9.1°) and MPB (21.3°, 30.7°, 34.8°, and 52.0°), indicating the successful combination of the two components. Taken together, the SEM and XRD results collectively demonstrate the successful construction of the MPB@ZnPc composite nanostructure.

These morphological changes indicate the successful integration of ZnPc and MPB via a polymer-templating strategy, with MPB predominantly decorating the surface of ZnPc.

The XRD patterns (Figure 1E) further confirmed the composite structure. Pure MPB exhibited characteristic diffraction peaks at 2θ = 21.3°, 30.7°, 34.8°, and 52.0°, while ZnPc showed peaks at 2θ = 6.9°, 9.1°, 12.5°, and 16.8°. Notably, MPB@ZnPc retained the characteristic peaks of both ZnPc and MPB without additional peaks, indicating successful physical combination of the two components. Collectively, the SEM and XRD results confirm the successful construction of the MPB@ZnPc composite nanostructure.

The N_2_ adsorption–desorption isotherms of MPB@ZnPc (Figure 1F) exhibited a combined type II/IV characteristic, consistent with its hybrid structure. The type II behavior is mainly attributed to the non-porous ZnPc matrix, while the type IV feature originates from the mesoporous MPB component. The hysteresis loop can be classified as H3-type, suggesting that the mesopores are mainly formed by interparticle voids between MPB nanoparticles and the ZnPc substrate.

The BET surface area of MPB was 57.89 m^2^/g with a pore volume of 0.071 cm^3^/g. After integration with ZnPc, the BET surface area of MPB@ZnPc decreased to 34.65 m^2^/g, accompanied by a reduction in pore volume, further confirming successful composite formation. The pore size distribution (Figure 1G) shows a distinct mesoporous peak for MPB, whereas MPB@ZnPc exhibits significantly reduced pore intensity, consistent with the BET results.

Figure 1H presents the zeta potential of MPB, ZnPc, and MPB@ZnPc. The zeta potential values were −38.70 ± 0.70 mV for MPB, −11.15 ± 0.65 mV for ZnPc, and −44.35 ± 0.95 mV for MPB@ZnPc. The noticeable shift in surface charge further confirms the successful recombination of ZnPc with MPB and the formation of a stable composite system.

### 2.2. Photothermal and Photocatalytic Performance

Based on the confirmed material structure, the photothermal and photocatalytic properties of MPB@ZnPc were further evaluated. The photothermal performance of MPB@ZnPc exhibited clear dependence on both concentration and laser power.

**Figure 2:**
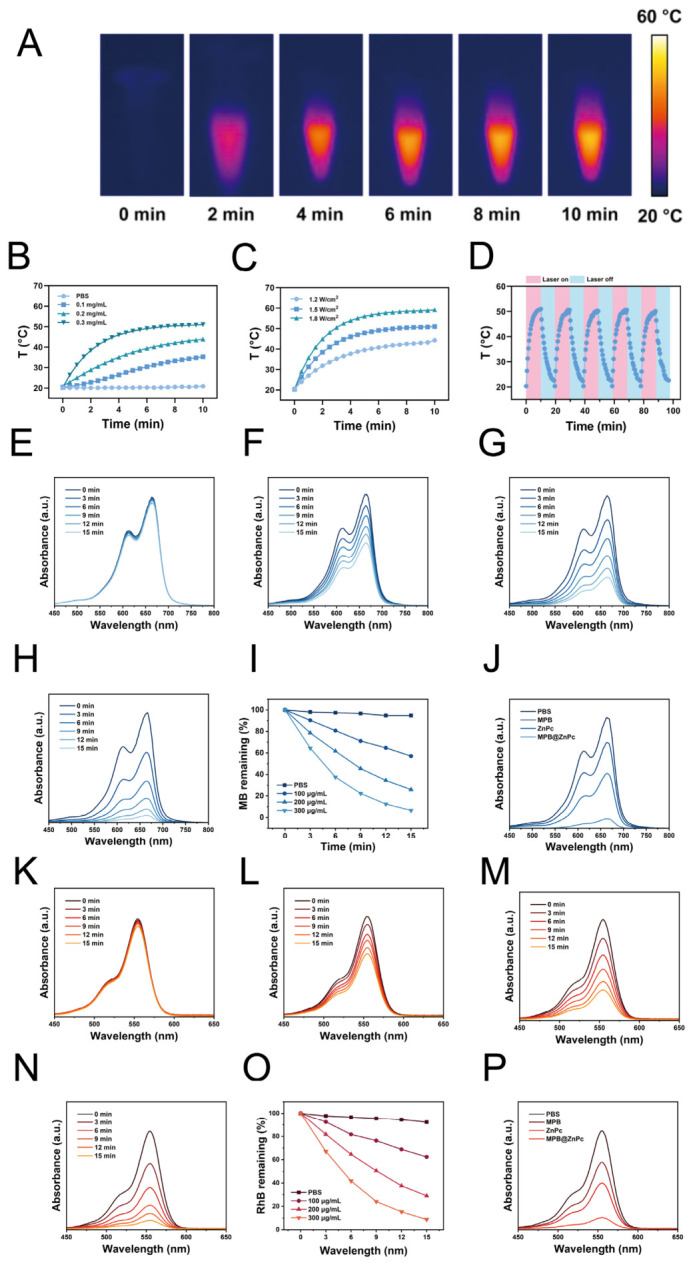
Photothermal and photocatalytic performance of MPB@ZnPc. (**A**) Infrared thermal images of MPB@ZnPc dispersion (0.3 mg/mL) under near-infrared (NIR) irradiation (1.5 W/cm^2^). (**B**) Temperature–time profiles of MPB@ZnPc dispersions at different concentrations under NIR irradiation (1.5 W/cm^2^). (**C**) Temperature time profiles of MPB@ZnPc dispersion (0.3 mg/mL) under different NIR power densities. (**D**) Photothermal stability of MPB@ZnPc over multiple heating cooling cycles. (**E**–**H**) Time-dependent UV–vis absorption spectra of methylene blue (MB) in the presence of PBS or MPB@ZnPc at different concentrations under NIR irradiation. (**I**) Time-dependent residual concentration of MB calculated from absorbance changes. (**J**) Comparative degradation of MB under different conditions (PBS, MPB, ZnPc, and MPB@ZnPc at 0.3 mg/mL) under NIR irradiation (1.5 W/cm^2^). (**K**–**N**) Time-dependent UV vis absorption spectra of rhodamine B (RhB) under different experimental conditions. (**O**) Time-dependent residual concentration of RhB. (**P**) Comparative degradation of RhB under PBS, MPB, ZnPc, and MPB@ZnPc under NIR irradiation (1.5 W/cm^2^). All experiments were performed in triplicate (n = 3).

As shown in Figure 2A, the temperature evolution of a 0.2 mg/mL MPB@ZnPc dispersion under 808 nm NIR irradiation (1.5 W/cm^2^) was recorded using a thermal imaging camera. The corresponding time–temperature profiles (Figure 2B) demonstrated that the temperature increased rapidly upon irradiation.

As illustrated in Figure 2B,C, the photothermal effect of MPB@ZnPc was strongly concentration-dependent. Under NIR irradiation at 1.5 W/cm^2^, dispersions with concentrations of 0.1, 0.2, and 0.3 mg/mL exhibited progressively enhanced heating performance, with final equilibrium temperatures increasing from 35.3 ± 0.3 °C to 51.0 ± 0.1 °C. Both the heating rate and maximum temperature increased with increasing concentration.

Similarly, the photothermal performance was dependent on laser power density. When irradiated at 1.2, 1.5, and 1.8 W/cm^2^, the temperatures reached 44.3 ± 0.2 °C, 51.0 ± 0.2 °C, and 59.2 ± 0.3 °C, respectively, indicating a positive correlation between temperature elevation and laser power.

The photothermal stability of MPB@ZnPc was further assessed through five on/off irradiation cycles (10 min irradiation followed by natural cooling). As shown in Figure 2D, the maximum temperatures (T_max) in each cycle were 51.0 ± 0.1 °C, 50.6 ± 0.2 °C, 50.2 ± 0.1 °C, 50.6 ± 0.3 °C, and 50.1 ± 0.2 °C, respectively. No significant decay in peak temperature was observed, and the heating–cooling curves overlapped well, indicating excellent photothermal stability.

The photocatalytic activity of MPB@ZnPc was evaluated by monitoring the degradation of methylene blue (MB) under NIR irradiation. As shown in Figure 2E–J, the degradation efficiency exhibited a clear concentration dependence, with removal efficiencies of 35.5 ± 0.4%, 67.3 ± 0.7%, and 95.2 ± 0.1% at concentrations of 0.1, 0.2, and 0.3 mg/mL, respectively.

Comparative experiments revealed that MPB@ZnPc exhibited superior photocatalytic performance compared to MPB and ZnPc alone, with ZnPc showing moderate activity. Similar results were obtained using rhodamine B (RhB) as the model substrate, further confirming the robust photocatalytic capability of the composite system.

### 2.3. In Vitro Antibacterial Activity

Following confirmation of the photothermal and photocatalytic properties, the in vitro antibacterial performance of MPB@ZnPc against *F. nucleatum* and *P. gingivalis* was evaluated using the colony-forming unit (CFU) method.

**Figure 3:**
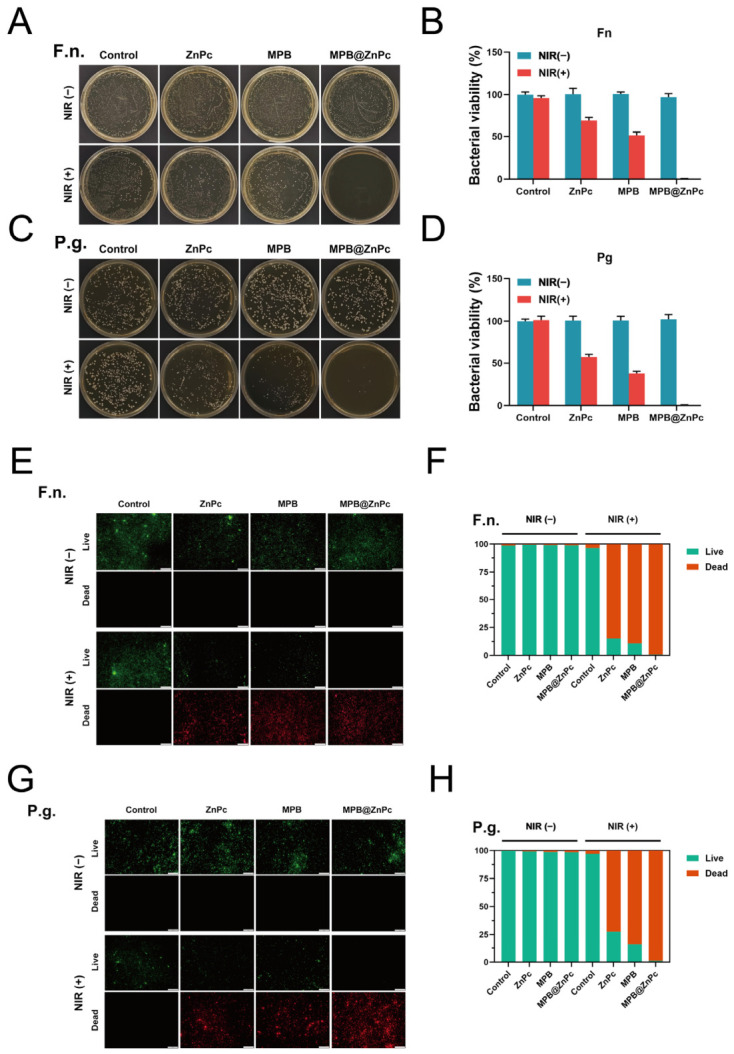
Antibacterial performance evaluated by CFU counting and live/dead staining. (**A**,**B**) CFU assay and corresponding quantification of *F. nucleatum* under different treatments. (**C**,**D**) CFU assay and corresponding quantification of *P. gingivalis* under different treatments. (**E**,**F**) Representative fluorescence images and quantitative analysis of live/dead staining for *F. nucleatum*. (**G**,**H**) Representative fluorescence images and quantitative analysis of live/dead staining for *P. gingivalis*. All experiments were performed in triplicate (n = 3).

As shown in Figure 3A–D, under dark conditions (NIR−), no significant differences in bacterial survival were observed among all groups compared with the PBS control, indicating that the materials exhibited negligible intrinsic antibacterial activity in the absence of irradiation.

Under NIR irradiation (NIR+), both MPB and ZnPc alone showed moderate antibacterial effects. Specifically, MPB reduced the survival rates of *F. nucleatum* and *P. gingivalis* to 37.83 ± 2.34% and 51.37 ± 3.83%, respectively, while ZnPc reduced them to 57.23 ± 3.20% and 68.67 ± 4.35%.

In contrast, MPB@ZnPc combined with NIR irradiation exhibited dramatically enhanced antibacterial activity, reducing the survival rates of *F. nucleatum* and *P. gingivalis* to 0.39 ± 0.52% and 0.23 ± 0.32%, respectively. This effect was significantly stronger than that of either MPB or ZnPc alone (*p* < 0.001), demonstrating a pronounced synergistic antibacterial effect.

Live/dead bacterial staining further confirmed these findings. As shown in Figure 3E–H, the PBS groups (with or without irradiation) predominantly exhibited strong green fluorescence, indicating high bacterial viability. In the MPB+NIR and ZnPc+NIR groups, both green (live) and red (dead) fluorescence signals were observed, suggesting partial bacterial killing.

Notably, the MPB@ZnPc+NIR group displayed predominantly red fluorescence with minimal green signal, indicating extensive bacterial death and severe disruption of bacterial integrity.

Collectively, these results demonstrate that MPB@ZnPc, under NIR irradiation, achieves highly efficient antibacterial activity through a synergistic mechanism.

### 2.4. Biofilm Removal and ROS Generation

**Figure 4:**
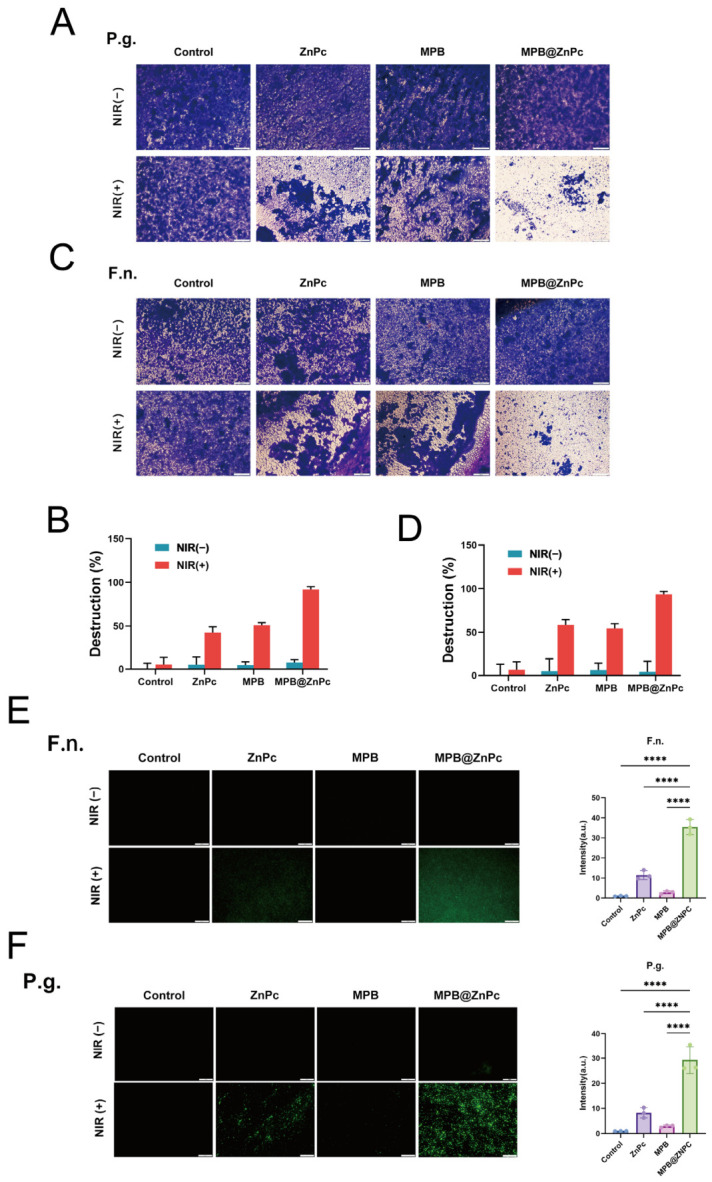
Biofilm removal and reactive oxygen species ROS generation of MPB@ZnPc. (**A**,**B**) Crystal violet staining images and quantitative analysis of biofilm removal for *Fusobacterium nucleatum*. (**C**,**D**) Crystal violet staining images and quantitative analysis of biofilm removal for *Porphyromonas gingivalis*. (**E**) Representative fluorescence images and quantitative analysis of intracellular ROS generation in *F. nucleatum* under different treatments, including statistical analysis of mean fluorescence intensity. (**F**) Representative fluorescence images and quantitative analysis of intracellular ROS generation in *P. gingivalis* under different treatments, including statistical analysis of mean fluorescence intensity. Statistical significance was determined using one-way ANOVA (**** *p* < 0.0001). All experiments were performed in triplicate (n = 3).

The biofilm removal capability of the materials was evaluated using crystal violet staining. Biofilm removal efficiency was calculated by normalizing the biofilm biomass of the NIR−group to 100%. As shown in Figure 4A–D, both MPB+NIR and ZnPc+NIR groups exhibited moderate biofilm disruption effects. For *Fusobacterium nucleatum*, the average removal rates were 51.10 ± 2.82% and 42.63 ± 6.58%, respectively, while for *Porphyromonas gingivalis*, the corresponding values were 54.57 ± 5.51% and 58.53 ± 6.09%, respectively.

In contrast, MPB@ZnPc combined with NIR irradiation achieved significantly enhanced biofilm eradication, with removal efficiencies of 93.25 ± 3.30% for *F. nucleatum* and 93.60 ± 3.30% for *P. gingivalis*. These results indicate that the composite nanomaterial exhibits markedly superior biofilm-disrupting capability compared with the individual components.

Intracellular reactive oxygen species (ROS) generation was further evaluated using the DCFH-DA fluorescent probe. As shown in Figure 4E,F, the MPB+NIR group exhibited weak and uneven green fluorescence, indicating limited ROS production. In contrast, the ZnPc+NIR group showed significantly stronger fluorescence, consistent with the intrinsic ROS-generating capability of ZnPc as a second-generation photosensitizer.

Notably, the MPB@ZnPc+NIR group exhibited the strongest and most uniform fluorescence signal, indicating substantial intracellular ROS accumulation. Quantitative analysis revealed that the mean fluorescence intensity in the MPB@ZnPc+NIR group was approximately 35-fold higher than that of the control in *F. nucleatum*, and 29-fold higher in *P. gingivalis*. These findings suggest that the synergistic interaction between photothermal and photodynamic effects in MPB@ZnPc significantly amplifies oxidative stress in bacteria, thereby enhancing antibacterial efficacy.

### 2.5. Biocompatibility and Anti-Inflammatory Effects Under Dark Conditions

**Figure 5:**
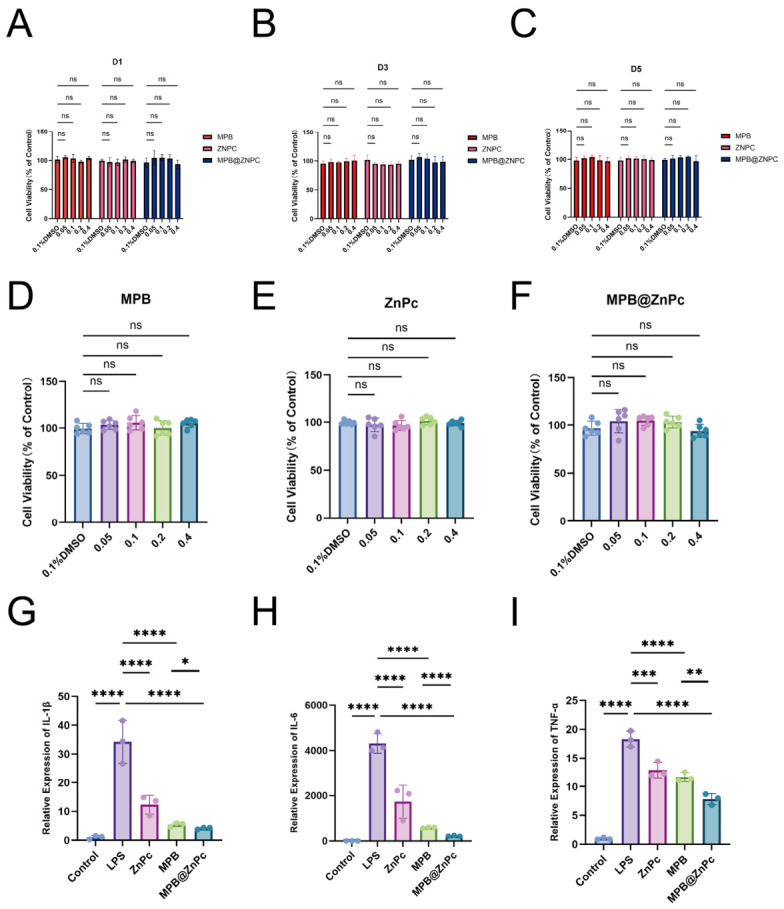
Biocompatibility and anti-inflammatory effects of the materials. (**A**–**C**) Cell viability of L929 fibroblasts after co-incubation with different concentrations of materials (0.05–0.4 mg/mL) for 1, 3, and 5 days, as evaluated by CCK-8 assay (n = 6). (**D**–**F**) Cytotoxicity of the materials toward RAW264.7 macrophages at different concentrations, assessed by CCK-8 assay (n = 6). (**G**–**I**) Relative mRNA expression levels of pro-inflammatory cytokines (*IL-1β*, *IL-6*, and *TNF-α*) in LPS-stimulated RAW264.7 cells treated with 0.3 mg/mL materials, as determined by qRT-PCR (n = 3). Statistical significance was determined using one-way ANOVA (* *p* < 0.05, ** *p* < 0.01, *** *p* < 0.001, **** *p* < 0.0001). All experiments were performed in triplicate (n = 3).

The biocompatibility of the nanomaterials was first evaluated using the CCK-8 assay. As shown in Figure 5A, MPB, ZnPc, and MPB@ZnPc exhibited negligible cytotoxicity toward L929 cells across a concentration range of 0.05–0.4 mg/mL over 1–5 days, indicating favorable time- and concentration-dependent biocompatibility.

Similarly, as shown in Figure 5B, no significant cytotoxicity was observed in RAW264.7 cells at concentrations below 0.4 mg/mL, supporting the suitability of these materials for subsequent cellular experiments.

To further investigate the anti-inflammatory effects, RAW264.7 cells were stimulated with lipopolysaccharide (LPS) and co-treated with different materials for 6 h. Total RNA was extracted and subjected to qRT-PCR analysis.

As shown in Figure 5C, under dark conditions (without NIR irradiation), MPB, ZnPc, and MPB@ZnPc all suppressed the transcription of pro-inflammatory cytokines, including *IL-1β*, *IL-6*, and *TNF-α*, to varying extents. Notably, MPB@ZnPc exhibited the most pronounced inhibitory effect compared to the individual components.

These results indicate that MPB@ZnPc possesses enhanced anti-inflammatory activity under dark conditions, suggesting its potential for modulating excessive immune responses associated with periodontitis.

### 2.6. Effects of MPB@ZnPc on Macrophage Polarization and NF-κB Signaling Pathway

Following the observed suppression of pro-inflammatory cytokine expression by MPB@ZnPc, its effects on macrophage polarization and the NF-κB signaling pathway were further investigated.

**Figure 6:**
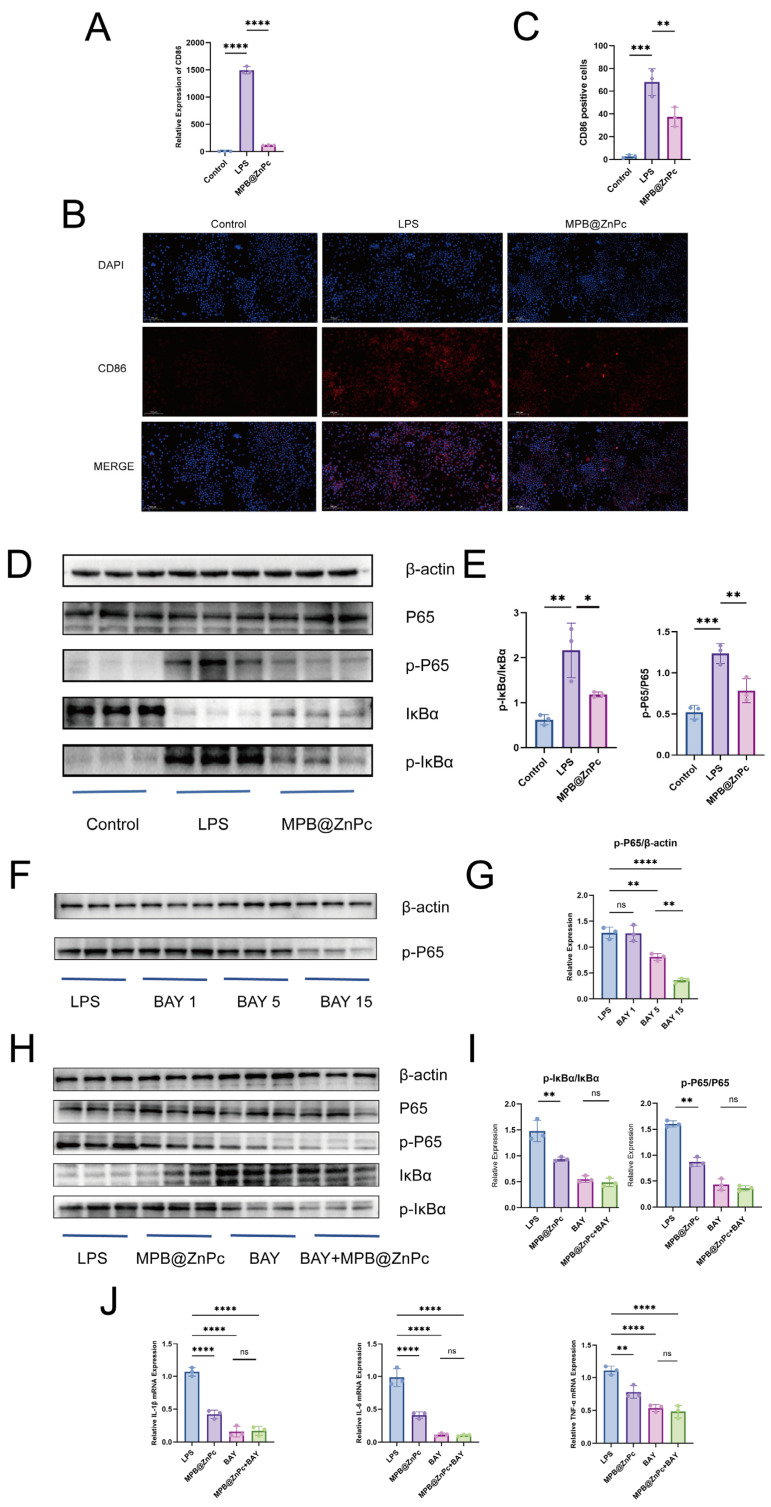
Effects of MPB@ZnPc on macrophage polarization and NF-κB signaling. (**A**) Relative mRNA expression of the M1 macrophage marker CD86 determined by qRT-PCR. (**B**,**C**) Immunofluorescence staining of CD86 in RAW264.7 cells and quantitative analysis of CD86-positive cells. (**D**) Expression and phosphorylation levels of NF-κB pathway-related proteins (IκBα and p65). (**E**) Quantitative analysis of Western blot band intensities. (**F**,**G**) Western blot analysis and densitometric quantification showing the inhibitory effects of different concentrations of BAY 11-7082 (1, 5, and 15μM) on LPS-induced NF-κB phosphorylation in RAW264.7 cells. (**H**,**I**) Western blot analysis and quantitative results comparing the effects of MPB@ZnPc, BAY 11-7082, and their combination on NF-κB pathway activation in LPS-stimulated RAW264.7 cells. (**J**) qRT-PCR analysis of pro-inflammatory cytokine expression (*IL-1β*, *IL-6*, and *TNF-α*) under different treatments (MPB@ZnPc, BAY 11-7082, and combination groups). Statistical significance was determined using one-way ANOVA (* *p* < 0.05, ** *p* < 0.01, *** *p* < 0.001, **** *p* < 0.0001). All experiments were performed in triplicate (n = 3).

RAW264.7 macrophages were stimulated with LPS and co-treated with MPB@ZnPc for 6 h. The mRNA expression of the M1 macrophage marker CD86 was then analyzed by qRT-PCR. As shown in Figure 6A, LPS stimulation significantly upregulated CD86 expression. In contrast, MPB@ZnPc treatment markedly reduced CD86 expression compared with the LPS group, indicating inhibition of M1 macrophage polarization.

Consistent results were obtained from immunofluorescence staining. As shown in Figure 6B,C, LPS stimulation led to a pronounced increase in CD86-positive (red fluorescence) cells, whereas MPB@ZnPc treatment significantly reduced the proportion of CD86-positive cells.

To further elucidate the underlying mechanism, key proteins in the NF-κB signaling pathway were evaluated by Western blotting. As shown in Figure 6D, LPS stimulation significantly increased the phosphorylation levels of IκBα and p65, indicating activation of the NFκB pathway. Notably, MPB@ZnPc treatment markedly reduced the phosphorylation levels of both IκBα and p65 compared with the LPS group. Quantitative densitometric analysis further confirmed these trends (Figure 6E).

To verify whether the anti-inflammatory effects of MPB@ZnPc are mediated through the NF-κB pathway, the specific NF-κB inhibitor BAY 11-7082 was employed. BAY 11-7082 is a well-known inhibitor that suppresses IκBα phosphorylation and NF-κB activation. A concentration gradient experiment revealed that 15 μM BAY 11-7082 most effectively inhibited p-p65 expression, and this concentration was used in subsequent experiments.

Four experimental groups were established: LPS, MPB@ZnPc, BAY 11-7082 (BAY), and MPB@ZnPc + BAY. Western blot analysis showed no significant difference in NF-κB pathway activation between the BAY group and the MPB@ZnPc + BAY group (Figure 6D,E), suggesting that MPB@ZnPc and BAY 11-7082 act on the same NF-κB signaling axis. Consistently, qRT-PCR results showed no significant differences in inflammatory cytokine expression between these two groups.

Collectively, these findings demonstrate that MPB@ZnPc effectively suppresses NF-κB pathway activation and inhibits M1 macrophage polarization under dark conditions, thereby exerting a potent anti-inflammatory effect.

## 3. Discussion

Periodontitis is a prevalent oral inflammatory disease characterized by a complex pathogenesis involving both microbial infection and host immune dysregulation [32]. Disease progression is driven primarily by two interrelated processes: the formation of pathogenic biofilms that promote bacterial persistence, and the excessive inflammatory response of the host to bacterial stimulation [33,34].

In this study, a multifunctional MPB@ZnPc nanoplatform was successfully constructed by integrating antibacterial photodynamic therapy (aPDT) and photothermal therapy (PTT), enabling synergistic antibacterial effects under near-infrared (NIR) irradiation, while simultaneously exerting immunomodulatory activity under dark conditions. The following discussion focuses on the synergistic antibacterial mechanism, immunoregulatory effects, and current limitations.

PTT and aPDT have been extensively explored as non-invasive antibacterial strategies. PTT relies on photothermal agents (PTAs) to convert light energy into heat, thereby inducing bacterial inactivation through localized hyperthermia [35]. However, many PTAs suffer from limited photothermal efficiency and potential thermal toxicity, which restrict their clinical translation [36]. Prussian blue (PB), an FDA-approved agent for treating thallium poisoning, exhibits excellent biocompatibility and efficient photothermal conversion under NIR irradiation [37,38]. Its mesoporous derivative (MPB) further increases surface area and loading capacity, making it a promising nanoplatform for multifunctional applications.

In parallel, aPDT utilizes photosensitizers (PSs) to generate reactive oxygen species (ROS) upon light activation [39]. However, conventional PSs often exhibit limited ROS yield and reduced efficacy in hypoxic microenvironments [36]. Zinc phthalocyanine (ZnPc), as a second-generation photosensitizer, demonstrates strong NIR absorption, high singlet oxygen quantum yield, and favorable biocompatibility under dark conditions [40].

Despite these advantages, both strategies have inherent limitations when used individually. PTT typically requires relatively high temperatures to achieve effective bacterial eradication, which may cause collateral damage to surrounding healthy tissues [41]. It has been reported that increasing the temperature from 50 °C to 55 °C can significantly exacerbate tissue injury and inflammatory responses [42]. In contrast, the therapeutic efficacy of aPDT is often compromised in hypoxic environments such as deep periodontal pockets [43,44].

Consistent with previous reports [45], our results demonstrate that combining PTT and aPDT can effectively overcome these limitations through synergistic interactions. Specifically, the localized hyperthermia generated by MPB enhances bacterial membrane permeability, facilitating ROS penetration produced by ZnPc. Meanwhile, elevated oxidative stress further sensitizes bacteria to thermal damage, enabling efficient antibacterial activity under relatively mild conditions. This synergistic mechanism is supported by the significantly enhanced bacterial killing, biofilm disruption, and ROS generation observed in the MPB@ZnPc system compared with its individual components.

Beyond antibacterial activity, excessive host inflammatory responses play a crucial role in periodontal tissue destruction [46,47]. However, most previous studies have primarily focused on antibacterial performance. For instance, the ICG-MXene system developed by Yu et al. [18] and the sPDMA@ICG nanoplatform reported by Shi et al. [48] demonstrated enhanced phototherapeutic antibacterial efficacy, but did not address host immune regulation. Similarly, although Wang et al. [49] developed a thermosensitive hydrogel with antibacterial and anti-inflammatory properties, its dependence on exogenous H_2_O_2_ limits clinical applicability and does not fully integrate PTT and aPDT within a single platform.

In contrast, the present study highlights the importance of simultaneously targeting bacterial infection and host immune dysregulation. Macrophage polarization plays a central role in inflammatory regulation, where excessive M1 polarization and overproduction of pro-inflammatory cytokines (*IL-1β*, *IL-6*, *TNF-α*) contribute to periodontal tissue destruction [21]. The NF-κB signaling pathway is a key regulator of inflammatory responses and is closely associated with periodontitis progression [27].

Our findings demonstrate that MPB@ZnPc significantly suppresses M1 macrophage polarization and downregulates pro-inflammatory cytokine expression under dark conditions, consistent with the intrinsic anti-inflammatory properties of both MPB and ZnPc [28,31]. Mechanistically, MPB@ZnPc inhibits the phosphorylation of IκBα and p65, thereby suppressing NF-κB pathway activation. These results suggest that the anti-inflammatory effects may arise from the combined immunomodulatory contributions of both components, leading to an amplified regulatory outcome.

Despite these promising results, several limitations should be acknowledged. First, the periodontal microenvironment is highly complex and involves diverse microbial communities. This study only evaluated representative pathogens (*P. gingivalis* and *F. nucleatum*), and single-species models cannot fully replicate the clinical oral microenvironment. Therefore, future studies will establish multispecies biofilm models to better simulate the complex oral ecosystem and systematically evaluate the antibacterial and biofilm-removal efficacy of MPB@ZnPc under more clinically relevant conditions. Second, the current study was mainly based on in vitro experiments, and in vivo evidence remains lacking. Future studies will further investigate the therapeutic efficacy of MPB@ZnPc in animal periodontitis models, including the evaluation of alveolar bone loss, periodontal inflammatory scores, major organ toxicity, and periodontal tissue regeneration. Third, the retention behavior, biodistribution, and long-term biosafety of MPB@ZnPc within periodontal tissues remain unclear. Further investigations involving in vivo tracking and chronic toxicity assessment are required to support clinical translation. Finally, although the in vitro cytotoxicity assays demonstrated good biocompatibility of MPB@ZnPc toward L929 and RAW264.7 cells under dark conditions, the potential effects of NIR irradiation on cell viability and surrounding tissues require further investigation.

In summary, MPB@ZnPc integrates synergistic PTT/aPDT antibacterial activity with intrinsic immunomodulatory effects under dark conditions, providing a promising strategy to simultaneously address biofilm persistence and excessive inflammation in periodontitis. This dual-functional nanoplatform offers a potential avenue for the development of precision therapeutics for periodontal disease.

## 4. Materials and Methods

### 4.1. Materials

Potassium ferricyanide (K_3_[Fe(CN)_6_]) was purchased from Aladdin Biochemical Technology Co., Ltd. (Shanghai, China). Polyvinylpyrrolidone (PVP), hydrochloric acid (HCl, 36.0–38.0%), and N,N-dimethylformamide (DMF) were obtained from Sinopharm Chemical Reagent Co., Ltd. (Shanghai, China).

*Porphyromonas gingivalis* (ATCC 33277) and *Fusobacterium nucleatum* (ATCC 25586) were obtained from the American Type Culture Collection (ATCC, Manassas, VA, USA). Brain heart infusion broth (BHI, 237500) and agar (BHI agar, 241830) were purchased from BD Difco (Franklin Lakes, NJ, USA).

RAW264.7 murine macrophages and L929 fibroblasts were purchased from Procell Life Science & Technology Co., Ltd. (Wuhan, China). Dulbecco’s Modified Eagle Medium (DMEM, 11965092), fetal bovine serum (FBS, 10099141), trypsin–EDTA solution (25200056), and penicillin–streptomycin solution (15140122) were obtained from Gibco (Grand Island, NY, USA). Primary antibodies against CD86 (83523-4-RR), IκBα (10268-1-AP), p-IκBα (82335-1-RR), p65 (80979-1-RR), and p-p65 (82335-1-RR), as well as goat anti-rabbit IgG (RGAR001) and goat anti-mouse IgG (RGAM001) secondary antibodies, were purchased from Proteintech (Wuhan, China).

Cell Counting Kit-8 (CCK-8) was obtained from GLPBIO (Montclair, CA, USA). RNA extraction reagent (FreeZol Reagent, R711) and cDNA synthesis kit (HiScript II Q Select RT SuperMix for qPCR, R233) were purchased from Vazyme (Nanjing, China). Reactive oxygen species (ROS) assay kit (S0033), BCA protein assay kit (P0012S), and bacterial live/dead staining kit (C2030S) were obtained from Beyotime Biotechnology (Shanghai, China). BAY 11-7082 (HY-13453) was purchased from MedChemExpress (Monmouth Junction, NJ, USA).

Zinc phthalocyanine (ZnPc, 341169), methylene blue (M9140), rhodamine B (R6626), and crystal violet (C0775), Triton X-100 (85111) were purchased from Sigma-Aldrich (St. Louis, MO, USA).

### 4.2. Synthesis of MPB@ZnPc Nanomaterials

ZnPc powder was first mixed with NaCl crystals at a mass ratio of 1:2 and thoroughly ground for 6 h. The resulting mixture was ultrasonically washed with deionized water, followed by vacuum filtration. This washing process was repeated four times to remove excess NaCl. The collected sample was then dried overnight in an oven at 80 °C.

Subsequently, the obtained ZnPc was dispersed in DMF, and an appropriate amount of PVP was added under stirring to form ZnPc@PVP. The product was collected by centrifugation, washed with ethanol and deionized water, and dried overnight at 70 °C.

Next, ZnPc@PVP was redispersed in deionized water, followed by the addition of K_3_[Fe(CN)_6_] and dilute HCl under continuous stirring. The reaction mixture was then transferred to an oven and maintained at 80 °C for 20 h to obtain ZnPc@PVP@MPB.

Finally, the product was thoroughly washed with ethanol and deionized water to remove the intercalated PVP, yielding the final MPB@ZnPc nanomaterial.

The synthesis method exhibited good reproducibility. Three independent batches showed relative standard deviations of less than 5% in morphology, particle size, and photothermal performance, with a stable yield above 85%.

### 4.3. Material Characterization

The morphology and microstructure of the samples were characterized using scanning electron microscopy (SEM, Hitachi S-4800) was purchased from Hitachi (Tokyo, Japan). X-ray diffraction (XRD) was purchased from Haoyuan Instrument Co., Ltd. (Dandong, China) was employed to determine the crystalline structure. The obtained diffraction patterns were compared with standard reference data to identify the phase composition and crystal structure. These characterization techniques collectively confirmed the successful synthesis and structural features of MPB@ZnPc.

The pore structure characteristics of the materials were further analyzed by nitrogen adsorption–desorption measurements using the Brunauer–Emmett–Teller (BET) method. Zeta potential analysis was performed to evaluate the surface charge properties of the materials. All experiments were independently repeated three times (n = 3).

### 4.4. Photothermal Performance Evaluation

MPB@ZnPc aqueous dispersions with different concentrations (0.1, 0.2, and 0.3 mg/mL) were prepared. Aliquots (200 μL) were irradiated with an 808 nm near-infrared (NIR) laser at a power density of 1.5 W/cm^2^ for 10 min. Temperature changes were recorded every 30 s using a thermal imaging camera, and time–temperature curves were plotted.

To further evaluate the effect of laser power, a 0.3 mg/mL dispersion was irradiated at power densities of 1.2, 1.5, and 1.8 W/cm^2^ for 10 min under identical conditions. In addition, five on/off irradiation cycles were conducted to assess photothermal stability. All photothermal experiments were independently repeated three times (n = 3). The distance between the laser source and the sample surface was fixed at 5 cm.

### 4.5. Photocatalytic Activity Evaluation

Methylene blue (MB) and rhodamine B (RhB) were used as model substrates to evaluate photocatalytic performance. PBS, MPB, ZnPc, and MPB@ZnPc were dispersed in MB or RhB aqueous solutions and irradiated with an 808 nm NIR laser at a fixed power density of 1.5 W/cm^2^. Samples were collected every 3 min, and absorption spectra were recorded using a UV–vis spectrophotometer.

In addition, MPB@ZnPc dispersions at different concentrations (0.1, 0.2, and 0.3 mg/mL) were tested under the same conditions. The residual concentrations of MB and RhB were calculated based on the changes in characteristic absorption peaks.

### 4.6. Bacterial and Cell Culture

*P. gingivalis* and *F. nucleatum* were cultured in brain heart infusion (BHI) broth under anaerobic conditions at 37 °C. Bacterial suspensions were collected at the logarithmic growth phase and adjusted to 1 × 10^8^ CFU/mL using PBS.

The complete culture medium consisted of 90% DMEM, 10% fetal bovine serum (FBS), and 1% penicillin–streptomycin. L929 and RAW264.7 cells were cultured in complete medium and passaged at 70–80% confluence. L929 cells were passaged using PBS washing and EDTA digestion, whereas RAW264.7 cells were passaged without enzymatic digestion.

### 4.7. In Vitro Antibacterial and Biofilm Removal Assays

Samples were divided into PBS, MPB, ZnPc, and MPB@ZnPc groups, each with and without NIR irradiation (NIR+ and NIR−). Antibacterial activity was evaluated using the colony-forming unit (CFU) counting method.

Briefly, 100 μL of bacterial suspension (1 × 10^8^ CFU/mL) was mixed with 100 μL of the corresponding treatment solution and incubated under anaerobic conditions at 37 °C for 1 h in the dark. The NIR+ groups were then irradiated with an 808 nm laser at 1.5 W/cm^2^ for 10 min, while the NIR− groups were kept in the dark. After treatment, samples were serially diluted, plated on BHI agar, and incubated anaerobically at 37 °C for 48 h before colony counting. Bacterial survival rate (%) was calculated as follows: Survival rate = (CFUtreatment/CFUcontrol) × 100%.

For biofilm assays, bacterial suspensions were seeded into 24-well plates and incubated for 48 h to allow mature biofilm formation. After removing planktonic bacteria by PBS washing, samples were treated as described above. Biofilms were fixed, stained with crystal violet (5 mg/mL), and quantified by measuring absorbance at 570 nm using a microplate reader. All antibacterial and biofilm removal experiments were independently repeated three times (n = 3).

### 4.8. Live/Dead Bacterial Staining and ROS Detection

Bacterial viability was evaluated using a commercial live/dead staining kit. DMAO (green fluorescence) and propidium iodide (PI, red fluorescence) were used to stain live and dead bacteria, respectively. After different treatments, bacterial suspensions were stained according to the manufacturer’s instructions and visualized using a fluorescence microscope. The ratio of dead to live bacteria was quantified based on fluorescence images.

Intracellular reactive oxygen species (ROS) levels were detected using the DCFH-DA probe. After treatment, DCFH-DA was added to the bacterial suspension at a final concentration of 10 μM, followed by incubation in the dark. Samples were subsequently exposed to NIR irradiation or kept under dark conditions as controls.

Fluorescence images were analyzed using ImageJ software (version 1.8.0) for quantitative evaluation. The mean fluorescence intensity was calculated using the following formula:

Mean fluorescence intensity (a.u.) = (integrated density of the region of interest − background integrated density)/area of the region of interest.

The fluorescence intensity of the control group (NIR− group) was normalized to 1.0, and all experimental data were expressed as relative values. All fluorescence-based experiments were independently performed in triplicate (n = 3).

### 4.9. Cell Viability Assay (CCK-8)

Cell viability was evaluated using the CCK-8 assay. L929 and RAW264.7 cells were seeded into 96-well plates at appropriate densities and treated with different concentrations of materials. After incubation, 10 μL of CCK-8 solution was added to each well containing 90 μL of medium and incubated for 3 h. Absorbance at 450 nm was measured using a microplate reader. All CCK-8 assays were independently repeated three times (n = 3).

Cell viability (%) was calculated as:

(Cell_treated − Cell_blank)/(Cell_control − Cell_blank) × 100%

### 4.10. RNA Extraction and qRT-PCR

RAW264.7 cells were divided into control, LPS, ZnPc, MPB, and MPB@ZnPc groups. Cells were seeded in 24-well plates (1 × 10^5^ cells/well) and treated as described. Total RNA was extracted using FreeZol reagent and reverse-transcribed into cDNA. Quantitative real-time PCR was performed using a QuantStudio 5 system (Thermo Fisher Scientific, USA). Gene expression levels were normalized to *ActB* and calculated using the 2^−ΔΔCt^ method. Primer sequences are listed in Table 1. All qRT-PCR experiments were independently repeated three times (n = 3).

### 4.11. Immunofluorescence Staining

Cells were seeded on coverslips and treated as described above. After fixation with 4% paraformaldehyde, permeabilization with Triton X-100, and blocking with 5% BSA, cells were incubated with primary antibody (CD86, 1:200) followed by fluorescent secondary antibody (1:300). Nuclei were stained with DAPI. Fluorescence images were acquired and CD86-positive cells were quantified. All immunofluorescence staining experiments were independently repeated three times (n = 3).

### 4.12. Western Blot Analysis

Cells were lysed using RIPA buffer supplemented with protease and phosphatase inhibitors. Protein concentrations were determined using a BCA assay. Equal amounts of protein were separated by SDS-PAGE and transferred onto PVDF membranes. After blocking, membranes were incubated with primary antibodies overnight at 4 °C, followed by secondary antibody incubation. Protein bands were visualized and analyzed using ImageJ software. All Western blot (WB) experiments were independently repeated three times (n = 3).

### 4.13. NF-κB Pathway Inhibition Assay

To investigate whether MPB@ZnPc exerts its anti-inflammatory effects through the NF-κB signaling pathway, the NF-κB-specific inhibitor BAY 11-7082 was used in this study. RAW264.7 cells were seeded into 6-well plates at a density of 5 × 10^5^ cells/well and cultured overnight. Cells were then co-treated with BAY 11-7082 at concentrations of 1, 5, and 15 μM together with 100 ng/mL LPS for 6 h. The protein expression level of p-p65 was subsequently evaluated by Western blot to determine the optimal inhibitor concentration.

All groups were stimulated with 100 ng/mL LPS for 6 h to induce inflammatory responses. The experimental groups were as follows: (1) LPS group (LPS only, vehicle control); (2) MPB@ZnPc group (LPS + 0.3 mg/mL MPB@ZnPc); (3) BAY group (LPS + 15 μM BAY 11-7082); and (4) MPB@ZnPc + BAY group (LPS + 0.3 mg/mL MPB@ZnPc + 15 μM BAY 11-7082). BAY 11-7082, MPB@ZnPc, and LPS were added simultaneously. After 6 h of incubation, cells were collected for subsequent analyses.

Western blot analysis was performed to detect key proteins involved in the NF-κB signaling pathway, including p-p65, p65, p-IκBα, and IκBα. In addition, qRT-PCR was conducted to evaluate the mRNA expression levels of the pro-inflammatory cytokines *IL-1β*, *IL-6*, and *TNF-α*.

### 4.14. Statistical Analysis

Statistical analysis was performed using GraphPad Prism 10 (GraphPad Software, USA). Data are presented as mean ± standard deviation (SD). Differences between groups were analyzed using Student’s *t*-test or one-way ANOVA followed by Tukey’s post hoc test. Statistical significance was defined as ns (not significant), * *p* < 0.05, ** *p* < 0.01, *** *p* < 0.001, and **** *p* < 0.0001.

## 5. Conclusions

In this study, a multifunctional MPB@ZnPc nanoplatform with both antibacterial and immunomodulatory properties was successfully developed. Under NIR irradiation, MPB@ZnPc achieved efficient eradication of key periodontal pathogens and effective biofilm disruption through the synergistic effects of photothermal therapy (PTT) and antibacterial photodynamic therapy (aPDT). In vitro assays demonstrated bactericidal rates of 99.61 ± 0.52% against *Porphyromonas gingivalis* and 99.77 ± 0.32% against *Fusobacterium nucleatum*, with corresponding biofilm removal rates of 93.60 ± 3.30% and 93.25 ± 3.30%, significantly higher than those of the individual components. Importantly, under dark conditions, MPB@ZnPc exhibited significant immunomodulatory activity by inhibiting NF-κB pathway activation, suppressing M1 macrophage polarization, and reducing pro-inflammatory cytokine expression. Collectively, these findings demonstrate that MPB@ZnPc offers a promising strategy for the synergistic management of bacterial infection and excessive inflammation in periodontitis, providing a potential platform for precision therapy.

## Figures and Tables

**Table 1:** qPCR primer sequences.

Target Gene	Forward Primer	Reverse Primer
*IL-1β*	TGCAGAGTTCCCCAACTGGTACATC	GTGCTGCCTAATGTCCCCTTGAATC
*IL-6*	CAACGATGATGCACTTGCAGA	GTGACTCCAGCTTATCTCTTGGT
*TNF-α*	ACCCTCACACTCACAAACCA	ATAGCAAATCGGCTGACGGT
*CD86*	CAGCACGGACTTGAACAACC	CTCCACGGAAACAGCATCTGA
*ActB*	CACTGTCGAGTCGCGTCC	TCATCCATGGCGAACTGGTG

The sequences of qPCR primers in the present study.

## Data Availability

Data are contained within the article.
